# *In Vitro* Roles of *Burkholderia* Intracellular Motility A (BimA) in Infection of Human Neuroblastoma Cell Line

**DOI:** 10.1128/spectrum.01320-23

**Published:** 2023-07-06

**Authors:** Niramol Jitprasutwit, Amporn Rungruengkitkun, Sanisa Lohitthai, Onrapak Reamtong, Nitaya Indrawattana, Nitat Sookrung, Thaniya Sricharunrat, Passanesh Sukphopetch, Narisara Chatratita, Pornpan Pumirat

**Affiliations:** a Center for Vaccine Development, Institute of Molecular Biosciences, Mahidol University, Nakhon Pathom, Thailand; b Department of Microbiology and Immunology, Faculty of Tropical Medicine, Mahidol University, Bangkok, Thailand; c Department of Molecular Tropical Medicine and Genetics, Faculty of Tropical Medicine, Mahidol University, Bangkok, Thailand; d Center of Research Excellence on Therapeutic Proteins and Antibody Engineering, Department of Parasitology, Faculty of Medicine Siriraj Hospital, Mahidol University, Bangkok, Thailand; e Biomedical Research Incubator Unit, Department of Research, Faculty of Medicine Siriraj Hospital, Mahidol University, Bangkok, Thailand; f Pathology and Forensic Science Department, Chulabhorn Hospital, Chulabhorn Royal Academy, Bangkok, Thailand; g Mahidol-Oxford Tropical Medicine Research Unit, Faculty of Tropical Medicine, Mahidol University, Bangkok, Thailand; South China Sea Institute of Oceanology

**Keywords:** actin-based motility, BimA, *Burkholderia pseudomallei*, human neuroblastoma cells, proteomics

## Abstract

The bacterial pathogen Burkholderia pseudomallei causes human melioidosis, which can infect the brain, leading to encephalitis and brain abscesses. Infection of the nervous system is a rare condition but is associated with an increased risk of mortality. *Burkholderia* intracellular motility A (BimA) was reported to play an important role in the invasion and infection of the central nervous system in a mouse model. Thus, to gain insight of the cellular mechanisms underlying the pathogenesis of neurological melioidosis, we explored the human neuronal proteomics to identify the host factors that are up- and downregulated during *Burkholderia* infection. When infected the SH-SY5Y cells with B. pseudomallei K96243 wild-type (WT), 194 host proteins showed a fold change of >2 compared with uninfected cells. Moreover, 123 proteins showed a fold change of >2 when infected with a knockout *bimA* mutant (Δ*bimA*) mutant compared with WT. The differentially expressed proteins were mainly associated with metabolic pathways and pathways linked to human diseases. Importantly, we observed the downregulation of proteins in the apoptosis and cytotoxicity pathway, and *in vitro* investigation with the Δ*bimA* mutant revealed the association of BimA with the induction of these pathways. Additionally, we disclosed that BimA was not required for invasion into the neuron cell line but was necessary for effective intracellular replication and multinucleated giant cell (MNGC) formation. These findings show the extraordinary capacity of B. pseudomallei in subverting and interfering with host cellular systems to establish infection and extend our understanding of B. pseudomallei BimA involvement in the pathogenesis of neurological melioidosis.

**IMPORTANCE** Neurological melioidosis, caused by Burkholderia pseudomallei, can result in severe neurological damage and enhance the mortality rate of melioidosis patients. We investigate the involvement of the virulent factor BimA, which mediates actin-based motility, in the intracellular infection of neuroblastoma SH-SY5Y cells. Using proteomics-based analysis, we provide a list of host factors exploited by B. pseudomallei. The expression level of selected downregulated proteins in neuron cells infected with the Δ*bimA* mutant was determined by quantitative reverse transcription-PCR and was consistent with our proteomic data. The role of BimA in the apoptosis and cytotoxicity of SH-SY5Y cells infected by B. pseudomallei was uncovered in this study. Additionally, our research demonstrates that BimA is required for successful intracellular survival and cell fusion upon infection of neuron cells. Our findings have significant implications for understanding the pathogenesis of B. pseudomallei infections and developing novel therapeutic strategies to combat this deadly disease.

## INTRODUCTION

Burkholderia pseudomallei is a Gram-negative facultative intracellular bacterium that causes melioidosis. This pathogen possesses one of the largest bacterial genomes and a large number of virulence factors, enabling its survival and pathogenesis in diverse and challenging environments (1, 2). Since it is intrinsically resistant to many antibiotics, a minimum of 6 months of eradication therapy is necessary (3, 4). B. pseudomallei infects and subsequently replicates inside both phagocytic and nonphagocytic mammalian host cells (5, 6). Furthermore, its ability to invade diverse cell types impairs host systems, including the central and peripheral nervous systems (7, 8). To survive inside the host cell, the B. pseudomallei Bsa type 3 secretion system (T3SS) plays a role in evading the endocytic vesicles and surviving in the cytoplasm (9). When B. pseudomallei enters the cytosol, *Burkholderia* intracellular motility A (BimA), induces actin polymerization to form actin tails (10). BimA is a member of the trimeric autotransporter family in B. pseudomallei that shares structural similarities with host Enabled/vasodilator stimulated phosphoprotein (Ena/VASP) actin polymerases (11, 12). BimA serves as an essential virulence factor of B. pseudomallei, playing a pivotal role in modulating the cytoskeleton through actin-based motility and facilitating intracellular movement within host cells. This process enables the bacterium to reach the host plasma membrane, promoting cell fusion and multinucleated giant cell (MNGC) formation, supporting disseminated infection, evading host immune defenses, and contributing to its pathogenicity (13). Although the precise function of BimA in the neuron cells requires further investigation, it has been reported that B. pseudomallei invades the central nervous system (CNS) via the trigeminal nerve in mice (7, 14, 15). Furthermore, *in vitro* assays have demonstrated that B. pseudomallei readily infects glial cells and a subpopulation of Schwann cells to form MNGCs (8).

Melioidosis is an infectious disease in humans that is endemic to Northern Australia and Southeast Asia (16). The number of melioidosis cases is increasing and is underestimated (17). Melioidosis occurs in almost every organ system, including the CNS, and presentation includes a wide range of clinical manifestations (16). Most patients with neurological melioidosis (neuromelioidosis) have encephalomyelitis and brain abscess with various degrees of involvement of the brainstem, frontal lobe of the cerebellum, and spinal cord (18, 19). CNS infection caused by B. pseudomallei is rare and was reported as the presenting symptom in only 3% of patients in the Darwin prospective melioidosis study in Australia and approximately 2% of patients with melioidosis in Southeast Asia (20, 21). Nonetheless, the mortality rate of patients with neurological melioidosis is 20% to 25% (18, 19). Moreover, long-term neurologic sequelae, including coordination and sensory deficits, have been reported in patients with confirmed neurological melioidosis (22). Approximately 36% of CNS melioidosis survivors are predicted to have functional and cognitive neurological impairment (23, 24).

B. pseudomallei isolates possess unique virulence factors that promote rapid dissemination to the CNS (23). We recently reported that cycle-inhibiting factor, a T3SS effector, plays a role in invading neuron cells (25). Geographically restricted variants of BimA are considered important factors that influence the outcome and clinical presentation of neurological melioidosis (26–28). Neurological melioidosis is more common in Australia than in Thailand (29), and there is a correlation between an allele of *bimA* and the clinical presentation and outcome of patients with CNS melioidosis (28, 30). B. pseudomallei isolated from the Northern Territory, Australia, revealed a geographically restricted subset that harbored a B. mallei-like *bimA* allele (*bimA_Bm_*). This variant is not found in Thailand, where B. pseudomallei isolates have a typical *bimA* allele (*bimA_Bp_*) (31). B. mallei is a clonal descendant of B. pseudomallei that lost metabolic capabilities essential for environmental survival. It is an obligate mammalian pathogen that causes glanders, which primarily affects solipeds (32). Although several studies have demonstrated the impact of BimA on neurological melioidosis, an *in vitro* investigation of B. pseudomallei-infected neuron cells has not been described (8, 33).

In this study, we used proteomic analysis and an *in vitro* infection assay of the immortalized human bone marrow-derived cell line SH-SY5Y to elucidate how BimA manipulates the host during neurological infection. We identified a number of up- and downregulated proteins that probably reflect the severity of neurological melioidosis. Furthermore, we examined the pathogenicity of a *bimA* knockout mutant (Δ*bimA*) and compared it with B. pseudomallei wild-type (WT) and a *bimA* complemented mutant (Δ*bimA*::*bimA*) and described their phenotypes in neuroblastoma cells.

## RESULTS

### Construction of a stable Δ*bimA* knockout mutant and a complemented strain of B. pseudomallei K96243.

To identify host factors exploited and regulated by B. pseudomallei BimA during neuron cell infection, we constructed a stable markerless *bimA* knockout mutant (Δ*bimA*) using a pEXKm5-based allele replacement method. The joined flanking sequence on both sides of *bimA* (approximately 400 bp each; see Fig. S1A in the supplemental material) was subcloned into pEXKm5 to delete the entire *bimA* gene from the chromosome of B. pseudomallei K96243. The absence of *bimA* was validated by PCR and DNA sequencing. Both B. pseudomallei K96243 WT and the Δ*bimA* knockout mutant underwent proteomic analysis.

Additionally, to evaluate the role of BimA in the pathogenesis of neuron cells infected by B. pseudomallei *in vitro*, a *bimA* complemented strain (Δ*bimA*::*bimA*) was generated by complementing the deleted region back into the Δ*bimA* mutant using the same vector (pEXKm5), as previously described. Because the sequence of *bimA* would be the same as the WT strain, we introduced silent mutations in *bimA* to differentiate between the WT and complemented strains (Fig. S1B). No differences in the growth rates of B. pseudomallei K96243 WT, Δ*bimA* mutant, and Δ*bimA*::*bimA* strains in nutritionally rich LB medium were observed (Fig. S2).

### Proteomic analysis of neuroblastoma cells infected with B. pseudomallei K96243 and Δ*bimA* knockout mutant.

**FIG 1:**
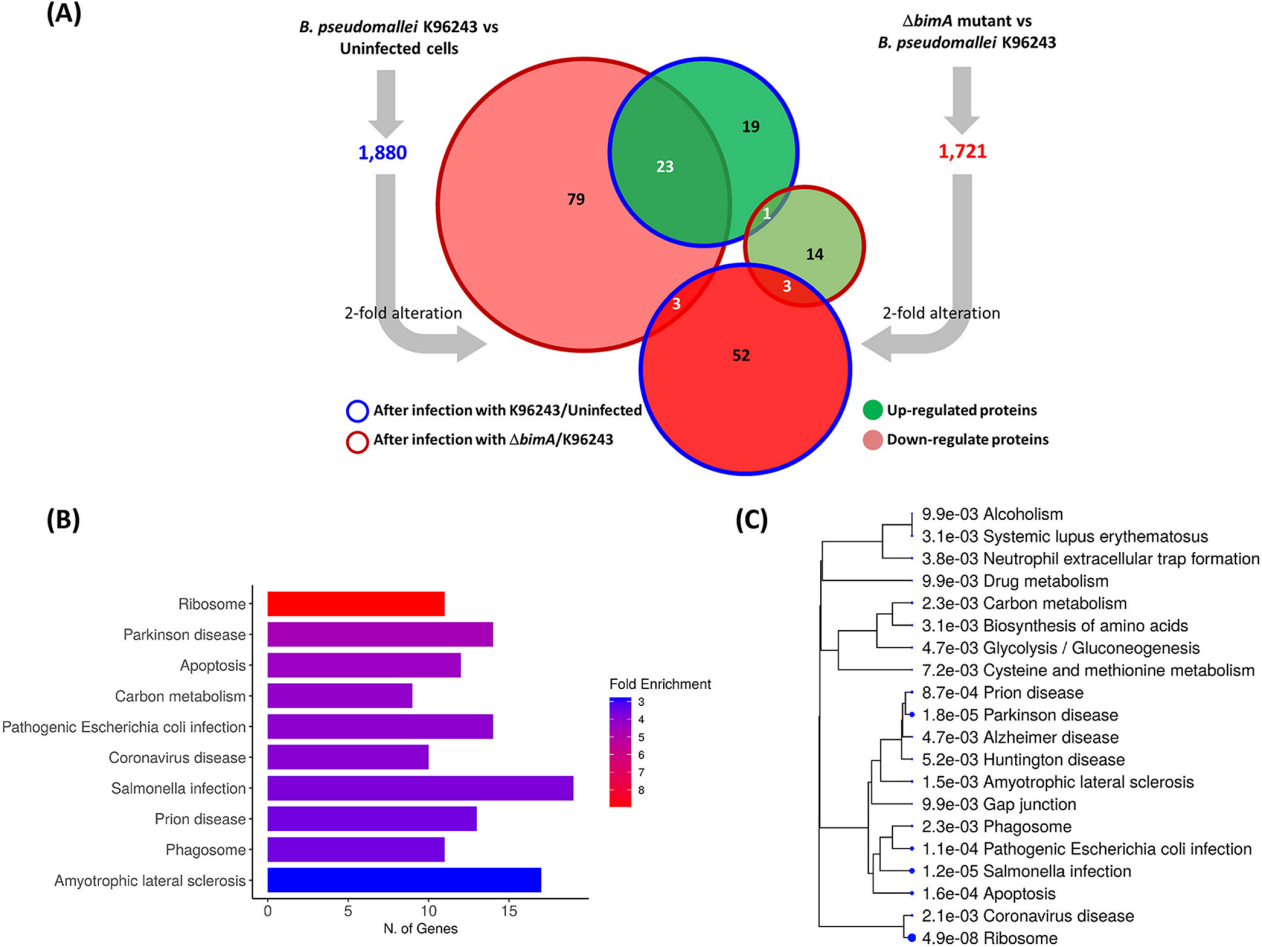
Analysis of proteomic profiles of the SH-SY5Y cell line infected with B. pseudomallei or the Δ*bimA* knockout mutant. (A) The Venn diagrams show the overlapping numbers of differential expression proteins in the SH-SY5Y cell line infected with B. pseudomallei K96243 compared to those in uninfected cells and the overlapping numbers of differential expression proteins in the SH-SY5Y cell line infected with the B. pseudomallei Δ*bimA* knockout mutant compared those in infected with B. pseudomallei K96243. (B) KEGG pathway enrichment analysis of all differentially expressed genes retrieved from proteomic profiling of a neuroblastoma cell line infected with B. pseudomallei K96243 and the Δ*bimA* knockout mutant showing the top 10 significantly enriched KEGG pathways. The most significant processes are shown in red, and the less significant processes are shown in blue according to fold enrichment values. (C) Hierarchical cluster tree summarizing the correlation among the 20 most significantly enriched KEGG pathways revealed after functional enrichment analysis of host cellular proteins in SH-SY5Y cell line infected with B. pseudomallei K96243 and the Δ*bimA* knockout mutant. The size of the solid blue dots corresponds to the enrichment false-discovery rate, with bigger dots indicating more significant *P* values. Pathways with many shared genes are clustered together. The graphical representations of the functional interactions based on KEGG pathways were generated using ShinyGO v0.741 (accessed 1 March 2023).

We utilized a proteomic approach to identify key proteins in human neuron cells underlying B. pseudomallei pathogenesis mediated by BimA. SH-SY5Y cells were subjected to infection by the B. pseudomallei WT and Δ*bimA* mutant strains. Protein profiling was performed in triplicate, and only the proteins detected in all replicates at a confidence level of 95% were subsequently identified. Then, significantly (>2-fold change) differentially expressed proteins (DEPs) were determined in neuron cells infected with B. pseudomallei K96243 compared with uninfected cells (K96243/Un) and in cells infected with the Δ*bimA* mutant compared with those infected with B. pseudomallei K96243 (Δ*bimA*/K96243). Pathway analysis of these proteins was performed using the Kyoto Encyclopedia of Genes and Genomes (KEGG) database. From a total of 1,880 proteins detected in neuron cells infected with B. pseudomallei WT, there were 101 DEPs in K96243/Un, comprising 43 upregulated and 58 downregulated DEPs (Fig. 1A and Table S1). On the other hand, a total of 1,721 neuron proteins were detected in Δ*bimA*/K96243 (Table S2), comprising 18 upregulated and 105 downregulated DEPs (Fig. 1A). We also identified 23 identical DEPs that were upregulated in K96243/Un and downregulated in Δ*bimA*/K96243 (Fig. 1A). Since these proteins were upregulated during infection with the WT strain, this suggests that they were induced by the indigenous virulence factor genes, including *bimA*. On the other hand, those proteins that were downregulated following infection with the Δ*bimA* mutant implied that their expression was reduced due to the absence of *bimA*. This suggests that BimA was associated with the induction of these 23 DEPs. Enrichment analysis revealed that these proteins were involved in metabolic pathways (e.g., carbohydrate and amino acid metabolism). Additionally, we detected three shared DEPs (histone H4, chimeric POTE-actin protein, and tubulin alpha-4A chain) that were downregulated in K96243/Un and upregulated in Δ*bimA*/K96243 (Fig. 1A). Since these proteins were downregulated following infection by the WT strain, this suggests that bacterial factors, including BimA, may suppress them. Furthermore, the proteins that were upregulated after infection with the Δ*bimA* mutant suggested that their expression was increased due to the absence of *bimA*. This implies that the presence of BimA negatively affects the expression of these three proteins, which are involved in homeostatic processes. Moreover, we identified a total of four DEPs that were shared between the K96243/Un and Δ*bimA*/K96243 groups. Three of these (beta-actin, histone H2B.1A [H2B1C], and neurogenic differentiation factor 6), were upregulated, while one DEP (histone H2B [H2B1B]) was downregulated in both the K96243/Un and Δ*bimA*/K96243 groups (Tables S1 and S2).

A total of 194 unique DEPs were further analyzed for putative functional roles by KEGG pathway analysis using the ShinyGO v0.741 enrichment analysis tool (34, 35). Among the top 10 enriched KEGG pathways (Fig. 1B), the greatest fold enrichment was clustered in the ribosome pathway, which is involved in processing genetic information. The second most significantly enriched pathway was that for amino acid biosynthesis. Moreover, the DEPs expressed during B. pseudomallei infection of neuron cells were significantly enriched in the apoptosis pathway (Fig. 1B). We also visualized relationships among these unique proteins using hierarchical clustering, which showed the 20 most significantly enriched functional categories in the KEGG pathways (Fig. 1C). The majority of enriched pathways were involved in metabolism. Importantly, the pathways for neurodegenerative disease clustered with those for bacterial infectious disease. Additionally, cellular processes, including cellular structures in eukaryotes (gap junction and tight junction) and cell growth and death (apoptosis) pathways, were grouped in the same cluster that was related to human diseases.

### BimA was involved in the cellular apoptosis of SH-SY5Y cells.

**FIG 2:**
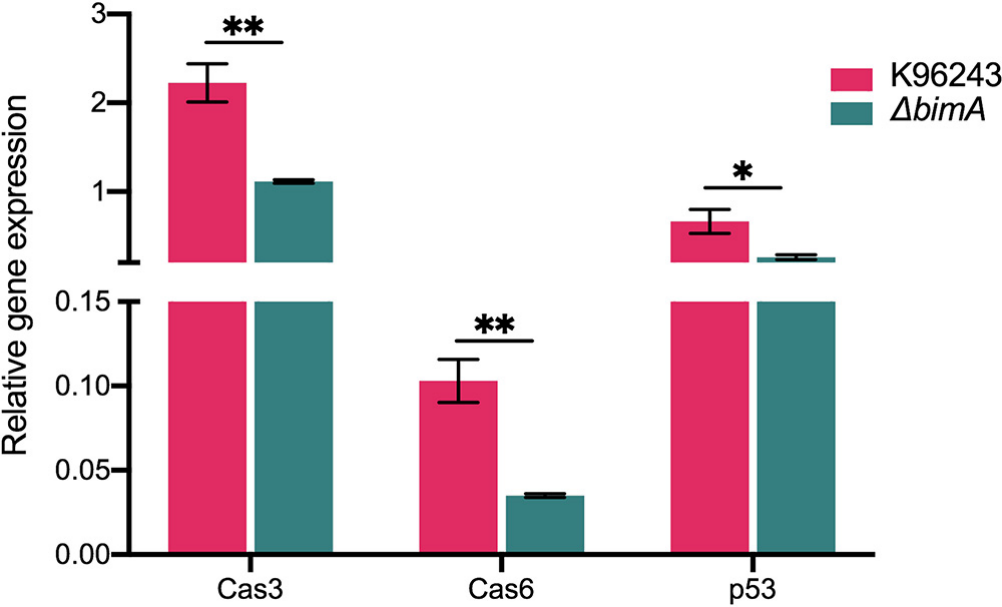
Different expression of cellular proteins after infection with B. pseudomallei K96243 or the Δ*bimA* knockout mutant. The relative gene expression over three biological replicates of Cas3, Cas6, and p53 at 10 h post-infection of the SH-SY5Y cell line by the B. pseudomallei WT or Δ*bimA* knockout mutant. Data represent the means ± SEM from three independent experiments. Student’s *t* test was performed using GraphPad Prism v8.0 software to calculate the *P* value; *, *P* < 0.05; **, *P* < 0.01.

The proteomic data revealed the association of BimA with the apoptosis pathway. Caspase-3 (encoded by CASP3) and caspase-6 (encoded by CASP6) proteins were downregulated in Δ*bimA*/K96243, with a 2.78- and 3.03-fold difference, respectively (Table S2). We also analyzed the expression of p53 tumor suppressor protein (encoded by TP53), which plays a crucial role in regulating apoptosis, to confirm involvement of the apoptosis pathway in neuron cell infection with B. pseudomallei. We determined the relative transcription level of these genes using quantitative reverse transcription PCR (qRT-PCR). Total RNA was extracted from the SH-SY5Y cells infected with B. pseudomallei K96243 or the Δ*bimA* mutant. The relative amount of mRNA transcription of all three genes in SH-SY5Y cells infected with Δ*bimA* mutant strains was lower than that in the cells infected with B. pseudomallei WT (Fig. 2). This finding was consistent with our proteomic analysis results, confirming the validity of the proteomic data in this study.

**FIG 3:**
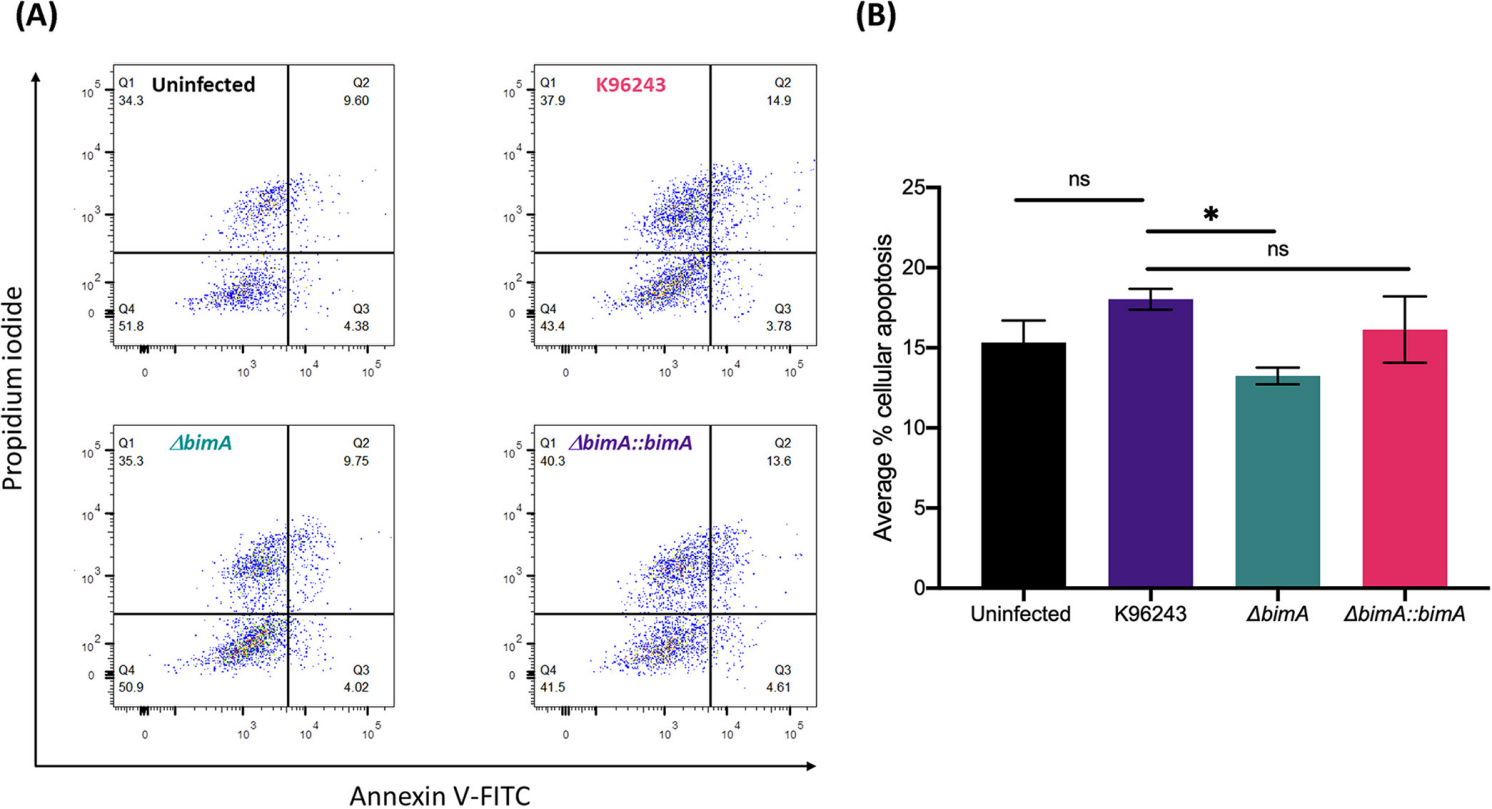
Detection of apoptosis by concurrent staining with annexin V and PI. (A) Representative flow cytometry results for apoptosis detected at 10 h in uninfected human SH-SY5Y neuroblastoma cells and after infection with by the B. pseudomallei WT or Δ*bimA* knockout mutant or Δ*bimA::bimA* complemented strain. (B) Percentages of cellular apoptotic cells. Data represent the means ± SEM from three independent experiments. Student’s *t* test was used to calculate the *P* value; *, *P* < 0.05; **, *P* < 0.01.

We constructed a *bimA* complemented mutant (Δ*bimA*::*bimA*) to clarify the role of BimA in the pathway of B. pseudomallei infection-induced apoptosis in neuron cells in three independent experiments. We used annexin V/propidium iodide (PI) staining and flow cytometry to investigate the effect of BimA on neuron cell apoptosis (Fig. 3A). B. pseudomallei K96243 induced early apoptosis of SH-SY5Y cells by 3.78% (quadrant 1: annexin V+/PI–) and late apoptosis by 14.90% (quadrant 2: annexin V+/PI–), in other words, a total of 18.68% of the apoptotic cells (quadrant 1 plus quadrant 2). After treatment with the Δ*bimA* mutant, the total percentage of cellular apoptosis was reduced to 13.77% (Fig. 3A). The average percentage of apoptotic cells among uninfected SH-SY5Y cells and SH-SY5Y cells infected with the Δ*bimA* mutant was 13.84% ± 0.14% and 13.25% ± 0.52%, respectively, which was a significant reduction from that of cells infected with B. pseudomallei K96243 (18.03% ± 0.65%). The SH-SY5Y cells infected with the Δ*bimA*::*bimA* strain recovered the average percentage of apoptotic cells to 16.14% ± 2.07% (Fig. 3B).

### Deletion of *bimA* reduced the cytotoxicity in neuroblastoma cells.

During apoptosis, the cytoplasmic enzyme lactate dehydrogenase (LDH) is released into the cell culture when the plasma membrane is damaged. Thus, the measurement of LDH release is often used as an indicator of apoptosis in *in vitro* settings. The proteomic data showed that LDH was present in cells infected with the B. pseudomallei WT and in cells infected with the Δ*bimA* mutant. LDH-A was upregulated in K96243/Un, with a fold change of 1.98. LDH-A, -B, and -C were downregulated in Δ*bimA*/K96243, with a fold change of 2.74, 2.62, and 2.91, respectively (Table S2).

**FIG 4:**
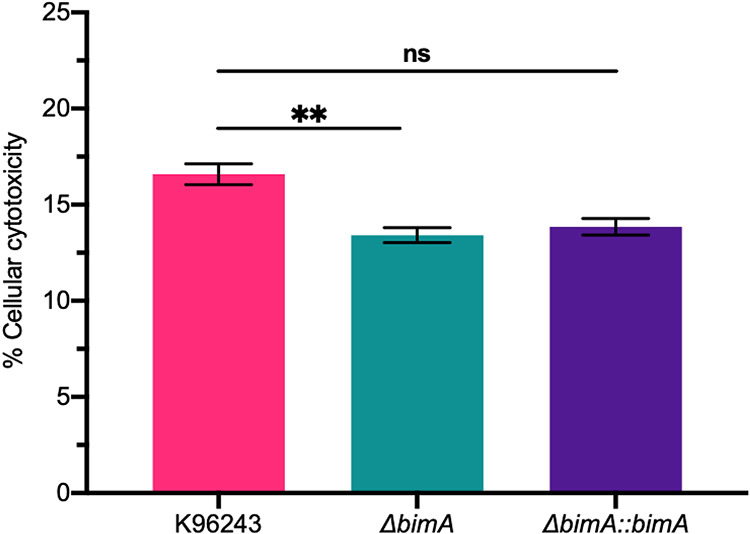
Cytotoxicity evaluation measured by LDH released from SH-SY5Y neuroblastoma cells infected by B. pseudomallei strains. The percentage of cellular cytotoxicity was quantified from the lactate dehydrogenase (LDH) release into the culture medium upon exposure to B. pseudomallei WT Δ*bimA* knockout mutant- or Δ*bimA::bimA* complemented strain-infected SH-SY5Y neuroblastoma cells for 10 h. Results are given in percentages related to uninfected control cells. Results are shown as the mean ± SEM values of three independent experiments. Student’s *t* test was used to calculate the *P* value; **, *P* < 0.01.

Next, we evaluated the level of LDH released from the lysates of infected cells to provide additional evidence of BimA mediating B. pseudomallei-induced apoptosis and to confirm the validity of the proteomic results. At 10 h post-infection, the extent of cell damage induced by the Δ*bimA* mutant strain was significantly lower than that induced by the WT strain (13.41% ± 0.387% and 16.59% ± 0.543%, respectively, *P* = 0.0414). When the cells were infected with the Δ*bimA*::*bimA* complemented strain, the percentage of cytotoxicity was 13.85% ± 0.430%, and there was no statistically significant difference compared with the WT strain (*P* = 0.0585; Fig. 4). Collectively, our findings suggest the involvement of BimA in B. pseudomallei-induced neuron cell apoptosis.

### B. pseudomallei *bimA* is unnecessary for invasion but is required for intracellular replication in neuroblastoma cells.

**FIG 5:**
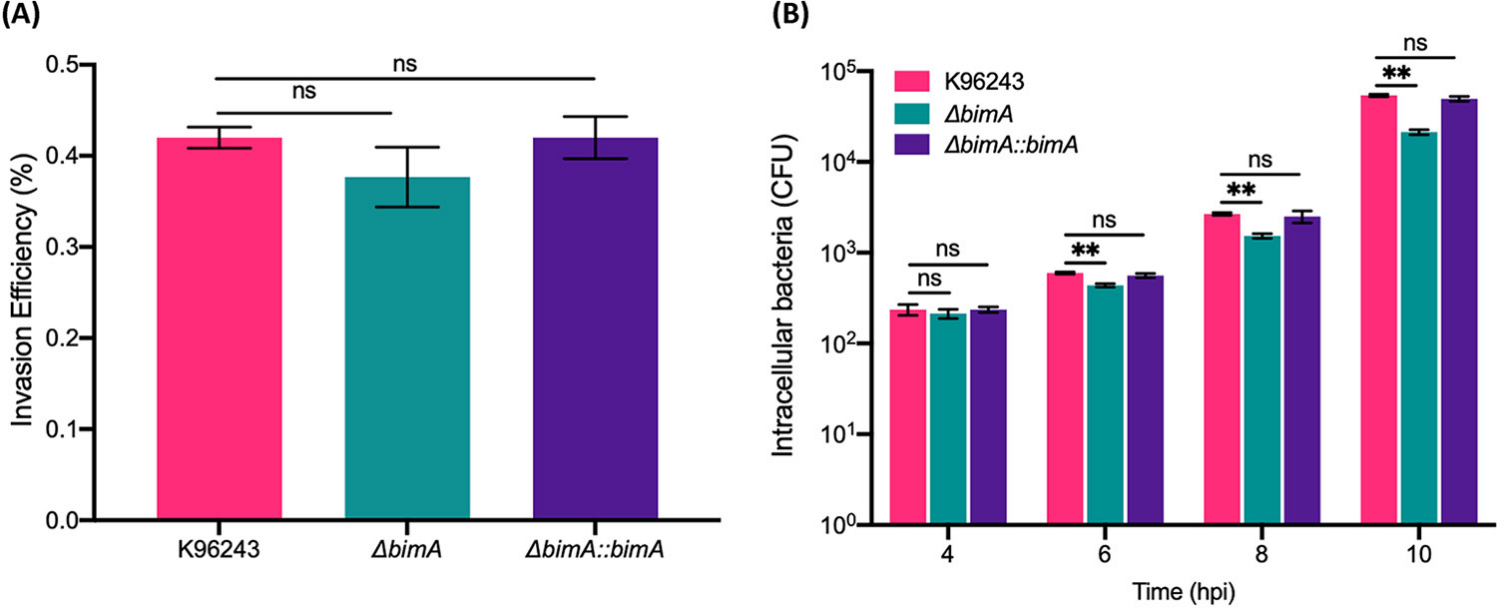
Invasion efficiency and intracellular survival of B. pseudomallei strains in human neuroblastoma SH-SY5Y cells. (A) Invasion of B. pseudomallei K96243, the *bimA* knockout mutant (Δ*bimA*), and the complemented *bimA* strain (Δ*bimA*::*bimA*) into SH-SY5Y cells. The invading bacteria were harvested at 3 h post-infection and plated on culture plates for enumeration. (B) Intracellular survival of B. pseudomallei K96243, the *bimA* knockout mutant (Δ*bimA*), and the complemented *bimA* strain (Δ*bimA*::*bimA*) at 4, 6, 8, and 10 h post-infection in SH-SY5Y cells. Data represent the means ± SEM from three independent experiments. Student’s *t* test was used to calculate the *P* value; **, *P* < 0.01.

To gain an overall view of how BimA mediates neuron cell infection, we performed *in vitro* infection assays using SH-SY5Y cells infected with B. pseudomallei WT and compared them with those infected with the Δ*bimA* mutant. The invasion efficiency of B. pseudomallei strains into neuroblastoma cells was 0.42% ± 0.012% and 0.377% ± 0.33% at 3 h post-infection for B. pseudomallei K96243 and the Δ*bimA* mutant, respectively (Fig. 5A). Thus, the absence of *bimA* did not impact the ability of B. pseudomallei to invade neuroblastoma cells (*P* = 0.2810). We performed the assay in parallel for Δ*bimA*::*bimA*, and the invasion efficiency was similar (0.42% ± 0.023%; Fig. 5A). In fact, all B. pseudomallei strains in this study exhibited comparable intracellular replication rates in neuron cells for up to 6 h post-infection, when a statistically significant reduction in the number of intracellular Δ*bimA* mutants was detected compared with the WT strain (*P* = 0.003; Fig. 5B). Nevertheless, an increased number of viable Δ*bimA* mutants was still observed at 8 and 10 h post-infection, but the viability of intracellular Δ*bimA* mutants was lower than that of the WT strain (Fig. 5B). These results suggested that while BimA may not be essential for invasion, it likely plays a role in the intracellular replication or survival of B. pseudomallei in neuron cells.

### The absence of *bimA* leads to defective cell-to-cell spread of B. pseudomallei in neuron cells.

The decreased number of intracellular Δ*bimA* mutants in neuron cells at 6 h post-infection could be due to the lack of actin-tail formation. It was reported that a *bimA* mutant escaped from late endosomes or lysosomes and was free in the cytoplasm at 3 h after inoculation in mouse macrophage J774.2 cells (10). Furthermore, the absence of actin tails formed by B. pseudomallei is a well-known consequence of BimA (10, 36). Giemsa staining of SH-SY5Y cells infected with B. pseudomallei showed the clustering of intracellular bacteria in cells infected with the Δ*bimA* mutant. In contrast, the bacteria in cells infected with the WT strain were spread more efficiently (Fig. S3). This Δ*bimA* mutant phenotype is consistent with previous studies (10, 36).

**FIG 6:**
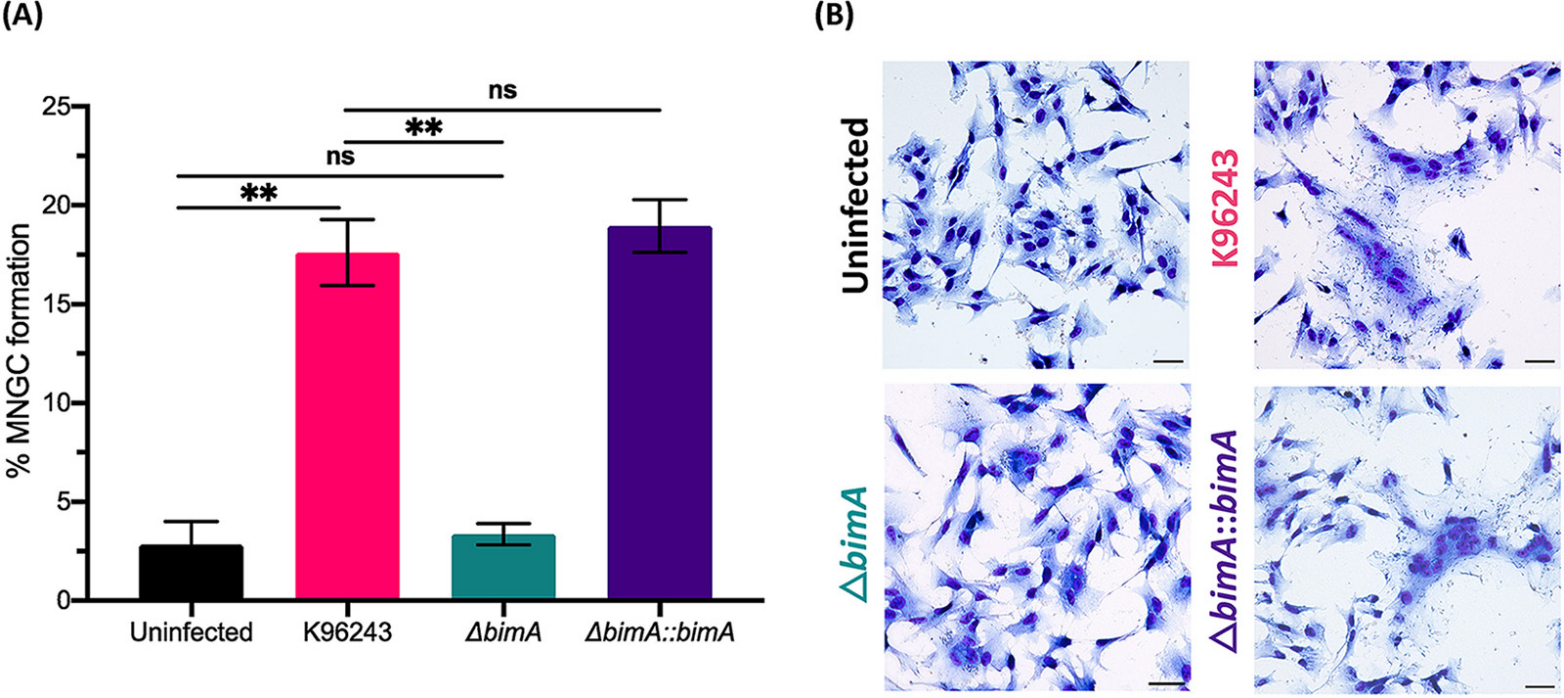
MNGC and plaque formation in SH-SY5Y cells induced by B. pseudomallei strains. (A) Percentage of MNGC formation induced by B. pseudomallei K96243, the *bimA* knockout mutant (Δ*bimA*), and the complemented *bimA* strain (Δ*bimA*::*bimA*); values are shown as the mean ± SEM of three independent experiments. **, *P* < 0.01. (B) Representative images of MNGC formation induced by B. pseudomallei K96243, the *bimA* knockout mutant (Δ*bimA*), and the complemented *bimA* strain (Δ*bimA*::*bimA*). Cells were stained with Giemsa and images were captured by microscopy with a 20× lens objective.

The involvement of actin-based motility in intercellular spread can be assessed according to the number of MNGCs. The number of MNGCs at 10 h post-infection with the Δ*bimA* mutant was dramatically decreased (3.36% ± 0.54%) compared with the WT strain (17.61% ± 1.6%, *P* = 0.0012) (Fig. 6A). This defect was restored by the complementation of *bimA*, and the percentage of MNGC formation of the Δ*bimA*::*bimA* complemented strain was 18.95% ± 1.337% (Fig. 6A). Notably, the free Δ*bimA* mutant bacteria replicated in the cytoplasm but restricted intracellular growth SH-SY5Y cells. However, the fusion of a minimum of three infected cells, which was defined as an MNGC, was observed in Δ*bimA* mutant-infected cells (Fig. 6B). In fact, MNCG formation was also detected in uninfected neuroblastoma cells (Fig. 6B), and the percentage of MNGC formation in the uninfected cells was similar to that of cells infected with the Δ*bimA* mutant (2.83% ± 1.171%; Fig. 6A). The number of MNGCs was substantially decreased in cells infected with the Δ*bimA* mutant, indicating that BimA played a major role in intercellular bacterial spread. The ability to spread into an adjacent cell was restored when *bimA* was complemented into the Δ*bimA* mutant (Fig. 6A). These results were consistent with those of the plaque formation assay assessed at 20 h post-infection, where the Δ*bimA* mutant showed a plaque formation defect in the SH-SY5Y neuroblastoma cell line (Fig. S4). Collectively, these findings demonstrate the involvement of BimA in intracellular replication and intercellular spread of B. pseudomallei in neuroblastoma cells.

## DISCUSSION

We identified DEPs that are associated with BimA and play an important role in neurological melioidosis using proteomic analysis to identify cellular proteins that are impacted by B. pseudomallei. We compared the DEPs of B. pseudomallei K96243-infected cells with uninfected cells (K96243/Un) and Δ*bimA* mutant-infected cells with B. pseudomallei K96243-infected cells (Δ*bimA*/K96243). The DEPs showed enrichment in pathways involved in infections and diseases, including Huntington disease and Alzheimer disease, and pathways of neurodegeneration, suggesting the involvement of BimA. Furthermore, this supports reports of the association of BimA with neurological melioidosis (26, 27). The involvement of host factors in the actin-based motility of B. pseudomallei was reported in proteomic data using BimA-expressing bacteria (37). As expected, actin-binding proteins, including alpha actin (acta1), gamma actin (actg1), tropomyosin 3 (tpm3), and 14-3-3 protein zeta/delta (ywhaz), that were downregulated in Δ*bimA*/K96243 in this study were also identified as recruited to BimA-expressing bacteria in previous work (37). Thus, these proteins are potential candidates for the treatment of melioidosis. For example, the expression of tropomyosins, which are involved in injury and neurological disease, in the CNS suggests their potential as a target for developing a future strategy resulting in complete neuronal repair after damage (38).

Our study used an *in vitro* cell culture infection assay to build up a more complete view of the infection of neuroblastoma cells by B. pseudomallei. We found that BimA was not involved in the invasion of neuroblastoma cells, which contradicts a previous study demonstrating the direct involvement of *bimA* in CNS invasion by B. pseudomallei MSHR520 (7). This could be due to the different settings of the infection experiments. In the aforementioned study, the Δ*bimA* mutant showed a defect in the invasion of the brain stem and spinal cord via the trigeminal nerve following intranasal inoculation, in which the olfactory bulb was a possible entry route for the pathogen (7). The olfactory bulb has a multilayered cellular structure, making it more complex than the cell culture environment. Although the previous study used B. pseudomallei MSHR520, which originated from Australia, K96243 possesses the same *bimA_Bp_* (31). B. pseudomallei strains isolated from Australian patients that harbor the *bimA_Bm_* allele impact the development and severity of CNS melioidosis (26, 27, 30). A study of the molecular mechanisms of the trimeric autotransporter BimA from B. pseudomallei and B. mallei showed that both use BimA to mimic the same cellular Ena/VASP protein to drive actin-based motility and dissemination in host cells (12). In neuron cells, harnessing the host cellular cytoskeleton, including Ena/VASP, is critical because it is important for actin dynamics and organization during nervous system development (39).

Although BimA did not affect the invasion of B. pseudomallei into neuroblastoma cells, it is necessary for intracellular replication. Our findings agree with a report of a reduced number of intracellular replications of an insertion BimA mutant in mouse J774.2 macrophage-like cells (40). A possible reason for this defect could be that the resources of nutrients and energy needed for the bacteria to survive and replicate become depleted. Failure to access a new intracellular niche may lead to the death of the bacteria or clearance by host cells. B. pseudomallei does not require cell protrusions; instead, cell fusion is crucial for forming MNGCs and intercellular spread (36, 41). Intracellular movement mediated by BimA enables the bacteria to propel to the cell membrane and consequently leads to cell contact, which is an essential condition for cell fusion. In agreement with our results and those of previous studies, B. pseudomallei lacking *bimA* showed defective MNGC and plaque formation (36, 42). Actin-based motility plays a crucial role in enabling dissemination within hosts and is essential for the survival of bacteria within host cells, leading to significant consequences. BimA is regulated by VirAG, a sensor-regulator that activates the transcription of the type VI secretion system (T6SS-1) (43). This suggests that BimA may have multiple effects in response to the changes during infection of mammalian hosts. Notably, the host cell actin-based motility machinery is also manipulated by Listeria monocytogenes, an intracellular Gram-positive bacterium that causes listeriosis. This pathogen spreads within axons to the brain and is driven by actin-based motility. Furthermore, most of the motile intracellular L. monocytogenes bacteria are present in neurofilament and axon-like processes in differentiated fetal bovine brain cells (44).

The use of differentiated SH-SY5Y cells with morphological characteristics of a mature neuronal-like phenotype enables further investigation of neurological melioidosis (45). Nonetheless, it is noteworthy that differentiation may enhance infectivity in this cell line, as demonstrated by a study of the infection of neuron cells with the Zika virus (46). However, the study focused on how the bacteria established an intracellular niche in neuron cells rather than neuronal function. Therefore, we used undifferentiated SH-SY5Y cells in this investigation.

Our study also explored the role of BimA in inducing cellular damage by measuring the release of LDH enzyme. It is noticeable that the Δ*bimA*::*bimA* strain induced cell damage at a similar level to that of the Δ*bimA* mutant, although there was no statistically significant difference of cellular cytotoxicity between the Δ*bimA*::*bimA* strain and either the WT or the Δ*bimA* mutant. A possible explanation for this outcome could be the synonymous codon usage bias. We introduced two silent mutations into the complemented strain to differentiate it from the WT using DNA sequencing. To achieve this, we integrated the full-length *bimA* gene with two single-nucleotide polymorphisms (SNPs) into the chromosome of the B. pseudomallei Δ*bimA* mutant strain through recombination at the homology regions, similar to the method used for generating the deletion mutant. Without these SNPs, it would be difficult to determine whether a strain is the WT or a complemented strain. However, this modification has the potential to impact protein expression levels, which could affect certain phenotypes. According to an analysis of B. pseudomallei codon usage frequency using an online codon statistics database (47), we found that replacing C with G in the ACC codon (which encodes threonine) transformed it into the preferred ACG codon from being less commonly used. Similarly, when C was replaced with T in the ATC codon (which encodes isoleucine), the commonly used ATC codon became the less preferred ATC codon. This alteration in codon usage may affect translation accuracy since preferred codons are known to be translated more precisely than to synonymous codons with lower frequency of usage (48). The effect of synonymous point mutations has been observed in a previous study that revealed that therapeutic X-ray doses caused synonymous mutations in the glucosyltransferases (*gtfs*) gene of Streptococcus mutans, resulting in reduced secretion of the GtfB enzyme without affecting bacterial growth, likely due to codon bias (49).

The correlation between MNGC formation and cytotoxicity during B. pseudomallei infection has been reported by demonstrating that a B. pseudomallei mutant confined within the cell cytoplasm (i.e., MNGCs were absent) was associated with a significant reduction in cytotoxicity in a human intestinal epithelial cell line (50). Another investigation of cell damage resulting in LDH release explained that host cell fusion coupled with genomic instability and the formation of nuclei induced by B. pseudomallei infection activates a signal pathway leading to a form of autophagic death and the release of LDH after the infection of macrophages (51). However, we did not detect proteins involved in the autophagic pathway in this study, and the discrepancies observed in the results may be attributed to the use of different cell types in the experiments. Since LDH release is an indicator of cell damage or death, we pursued a more specific investigation into apoptosis. B. pseudomallei-induced apoptosis in both phagocytic and nonphagocytic cells (52). In our study, proteomic profiling identified DEPs associated with the apoptosis pathway. qRT-PCR confirmed that caspase-3 and caspase-6 expression was reduced during SH-SY5Y cell infection with the Δ*bimA* mutant. Caspase-3 and Bax, which is a proapoptotic protein, are key determinants in the p53 apoptotic signaling cascade in neuronal cell death following injury (53). Accordingly, we also included the tumor protein p53, a key regulator of cell cycle arrest and apoptosis, in this study. Although p53 was not detected in our proteomic data, qRT-PCR showed a lower level of gene expression induced by the Δ*bimA* mutant. The rates of p53 translation and degradation in neuroblastoma cells may be the cause of this inconsistent outcome. The level of p53 expression influences its target genes and subsequent cellular responses, leading to changes in levels over time (54).

We postulated that neuron cells undergo apoptosis in response to B. pseudomallei infection to aid in the spread of infection. To validate the association of BimA-induced apoptosis with neurological melioidosis, we performed a cytometry-based assay for apoptosis. We found that the average percentage of apoptotic cells was reduced in SH-SY5Y cells infected with the Δ*bimA* mutant, and the complementation of *bimA* reversed this defect. Moreover, the reduction of cell damage in neuron cells infected by the Δ*bimA* mutant mirrors the decreased level of LDH expression, in accordance with our proteomic data. This phenotype appears to be specific to neuron cells because the activation of apoptotic caspase-3 does not mediate the cell damage observed in human hepatocyte HepG2 cells infected with B. pseudomallei (55). These findings suggest that the pathogenic mechanisms of B. pseudomallei may differ across cell types, highlighting the importance of investigating cell-specific responses to infection. Further comprehensive study is warranted.

**FIG 7:**
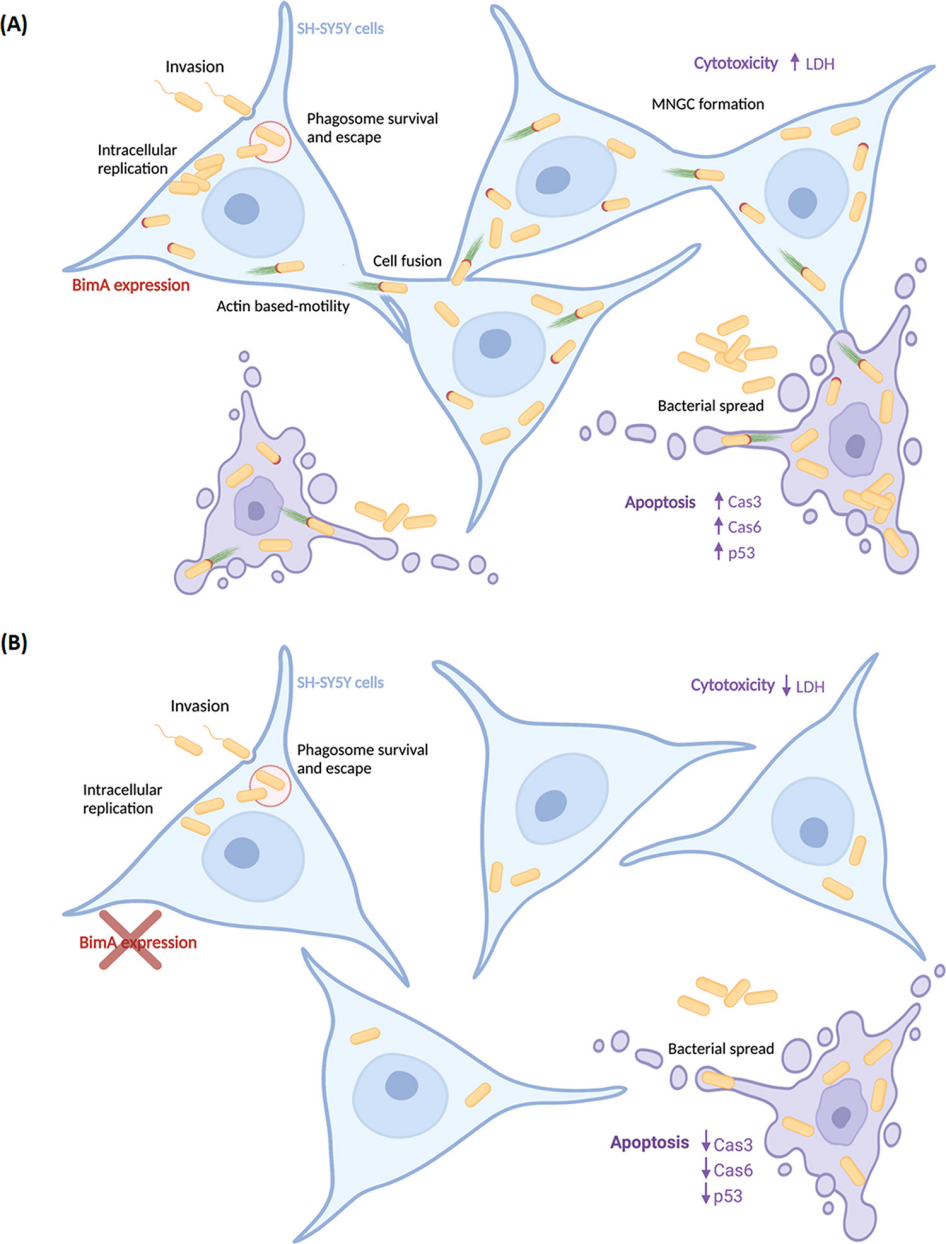
Proposed model of BimA-mediated intracellular life cycle of B. pseudomallei in human neuroblastoma SH-SY5Y cells. (A) Intracellular invasion and dissemination of the B. pseudomallei WT in SH-SY5Y cells. After invading, the bacteria escape into the cytoplasm, multiply, and induce expression of BimA to promote actin-based motility and cell-to-cell fusion, which ultimately lead to MNGC formation. This process coordinates the induction of the cytotoxicity and apoptosis of host cells. (B) Defective intracellular survival of the B. pseudomallei *bimA* mutant in SH-SY5Y cells. A deletion mutant of B. pseudomallei *bimA* can invade the SH-Sy5y cells and undergo intracellular replication. However, deletion of BimA causes the defect of intracellular survival and spreading of B. pseudomallei in human neuroblastoma SH-SY5Y cells, which concordantly influences the decrease of the cytotoxicity and apoptosis of host cells.

In conclusion, the identification of proteins from our proteomic data, combined with the characterization of the Δ*bimA* mutant in neuron cells, has the potential to enhance the understanding of neurological melioidosis and aid the development of targeted treatments. We demonstrated the various possible roles of BimA during neurological infection caused by B. pseudomallei (Fig. 7). Consistent with previous studies, we found that BimA was associated with cytoskeleton proteins and played a crucial role in the intra- and intercellular spread of the bacteria. Regulatory protein factors of the actin cytoskeleton, such as gamma actin, are involved in early neuronal differentiation resulting in different morphologies (56). We suggested that B. pseudomallei harnessed the machinery of actin-based motility mediated by BimA to reorganize the cell structure, thereby facilitating cell-to-cell spread. Furthermore, we proposed the novel function of BimA in regulating apoptosis and cellular toxicity during neuron infection (Fig. 7). Our findings offer supplementary proof of how this pathogen exploits cellular systems to establish infection and the importance of BimA in the progression of neurological melioidosis. This research will help advance the functional proteomic analysis of BimA and the understanding of its effects on B. pseudomallei pathogenicity. We believe that elucidation of the *in vitro* neurological pathogenesis of B. pseudomallei will not only decipher the molecular functions of B. pseudomallei effector BimA but also contribute to a comprehensive understanding of neuron cells.

## MATERIALS AND METHODS

### Bacterial strains, cell lines, and growth conditions.

The reference strain used in our study was B. pseudomallei K96243 WT, which was obtained from a melioidosis patient in northeastern Thailand. The *bimA* knockout mutant (Δ*bimA*) and the *bimA* complemented mutant (Δ*bimA*::*bimA*) strains were constructed as described below. Escherichia coli strains DH5α and RHO3 were used for cloning and conjugation of the delivery plasmid into B. pseudomallei for the generation of mutant and complemented strains, respectively. All bacterial strains were cultured in Luria-Bertani (LB) medium at 37°C with shaking at 200 rpm, with the exception of E. coli RHO3, which was cultured in LB medium supplemented with 400 μg/mL 2,6-diaminopimelic acid.

The human neuroblastoma SH-SY5Y cell line (CRL-2266; ATCC) was maintained in Dulbecco modified Eagle medium (DMEM; Gibco BRL) supplemented with 10% (vol/vol) heat-inactivated fetal bovine serum (Gibco BRL) and penicillin-streptomycin solution (Gibco BRL) (57). The cells were cultured at 37°C in a 90% humidity-controlled incubator with 5% CO_2_, and the culture medium was replaced every 2 days. When the cells reached approximately 90% confluence, they were detached from the surface of cell culture flasks using a 0.25% (wt/vol) trypsin-EDTA solution.

### Construction of the B. pseudomallei *bimA* knockout mutant and the complemented strain.

Deletion and complementation of the *bimA* gene in the B. pseudomallei K96243 genome (locus tag BPS_RS32200, old locus tag BPSS1492) were performed using a markerless allele replacement method with the pEXKm5 suicide vector (58). The primer sequences (Table S3) were designed using the National Center for Biotechnology Information (NCBI) Primer-BLAST tool (http://www.ncbi.nlm.nih.gov/tools/primer-blast). Briefly, the 5′ upstream and 3′ downstream fragments of *bimA* were amplified using primer pair F1-BimA-NotI and R1-BimA and primer pair F2-BimA and R2-BimA-XhoI, respectively. The resultant fragments were subjected to splicing by overlap extension PCR (SOEing PCR) using the primers F1-BimA-NotI and R2-BimA-XhoI. Then, the PCR amplicon with a deletion in the *bimA* region that was flanked by restriction sites for NotI and XhoI was ligated with linearized pEXKm5 digested with the same enzymes and transformed into E. coli DH5α cells. The obtained clones were validated by colony PCR using the primers for SOEing. The plasmid was extracted from positive clones and validated by DNA sequencing, followed by retransformation into E. coli RHO3, which was used for plasmid delivery to the host B. pseudomallei K96243 by conjugation, resulting in the creation of merodiploid strains. Kanamycin (1,000 μg/mL) was used for conjugant selection, which was then confirmed by PCR using primers flanking the mutant allele (F1-BimA-NotI and R2-BimA-XhoI). For merodiploid resolution using sucrose selection, the positive conjugants were streaked onto yeast extract tryptone (YT) agar (yeast extract and tryptone, BD; agar, Oxoid) containing 15% (wt/vol) sucrose (YTS) and incubated at room temperature for 72 h. The resultant single colonies were purified by restreaking on YTS plates and selected for kanamycin-sensitive clones. Primers designed to target the oriT region of pEXKm5 were used to ensure that the plasmid was absent from the mutant (59).

The Δ*bimA*::*bimA* strain was generated using the allele replacement method, as described above. Additionally, single-nucleotide polymorphisms (SNPs) that did not result in an amino acid change (silent mutations) were created to distinguish the WT and complemented strains: G was substituted for C at position 618 and T for C at position 621 of *bimA*, which did not change the corresponding amino acids (threonine and isoleucine, respectively). The left fragment containing the upstream and the 5′ region of *bimA* was amplified by PCR using F1-BimA-NotI and R1-BimA-SNP. The right fragment containing the downstream and the 3′ region of *bimA* was amplified by PCR using F2-BimA-SNP and R2-BimA-XhoI. The result of the deletion and complementation of *bimA* was validated by PCR using primers flanking the targeted region (Seq-F-BimA and Seq-R-BimA) and confirmed by DNA sequencing.

### Proteomic study.

SH-SY5Y cells were infected with B. pseudomallei strains, as described previously (25). Briefly, the SH-SY5Y cells were seeded at a density of 5 × 10^4^ cells per well in a 24-well cell culture plate. The following day, the medium was removed and replaced with fresh antibiotic-free DMEM. The SH-SY5Y cells were infected with the overnight cultures of B. pseudomallei strains at a multiplicity of infection (MOI) of 20. B. pseudomallei WT and Δ*bimA* mutant strains, as well as uninfected cells, were used for the proteomic assay. At 2 h post-infection, the extracellular bacteria were killed by adding 500 μL of fresh DMEM containing 250 μg/mL kanamycin (Sigma) and incubated at 37°C for another 8 h. Thereafter (10 h post-infection), the infected SH-SY5Y cells were lysed with lysis buffer containing 1% (vol/vol) sodium dodecyl sulfate, 1% (vol/vol) Triton X-100, and 0.5% (vol/vol) sodium chloride. Total cell lysates of B. pseudomallei-infected SH-SY5Y cells were used for proteomic analysis.

The protein concentration was measured using a Quick Start Bradford protein assay kit (Bio-Rad Laboratories). Bovine serum albumin was used as the protein standard. A total of 30 μg protein of each sample was separated using 12% sodium dodecyl sulfate polyacrylamide gel electrophoresis. The bands were visualized using Coomassie blue G (Merck) staining. Then, each lane was excised and cut into small pieces. For in-gel digestion, the gel pieces were destained using 50% (vol/vol) acetonitrile in 50 mM ammonium bicarbonate. Next, the pieces were reduced in 4 mM dithiothreitol (DTT) at 60°C for 15 min and alkylated by adding 250 mM iodoacetamide at room temperature in the dark for 30 min. The reaction was quenched with 4 mM DTT and dehydrated in 100% acetonitrile. The gel pieces were then rehydrated with 10 ng/μL trypsin in 50 mM ammonium bicarbonate at 37°C overnight. Finally, 100% acetonitrile was added to extract the peptides. The supernatant was collected, and the peptide mixtures were completely dried with a SpeedVac concentrator (Eppendorf, Hamburg, Germany).

Trypsin-digested samples were resuspended in 0.1% (vol/vol) formic acid containing 2% (vol/vol) acetonitrile and then analyzed using an UltiMate 3000 Nano LC system (Dionex, Surrey, UK) coupled with a micrOTOF-Q mass spectrometer (Bruker Daltonics, Bremen, Germany) to analyze the digested proteins. Data acquisitions were controlled using HyStar software (Bruker Daltonics, Bremen, Germany). MS and MS/MS spectra covered the mass range of *m/z* 400 to 2,000 and *m/z* 50 to 1,500, respectively. LC-MS/MS data files were searched using Mascot v2.4.1 (Matrix Science, London, UK) against the NCBI database. Protein identification was accepted at a confidence level of 95%. Semiquantitative analysis of the protein abundance was performed using the exponentially modified protein abundance index. The statistical significance (*t* test, *P* = 0.05) determined by the Perseus software platform was used to generate a volcano plot.

### Quantitative reverse transcription-PCR (qRT-PCR).

Total RNA was extracted from the SH-SY5Y cells infected with B. pseudomallei strains using an RNeasy minikit (Qiagen) and treated with RNase-free DNase I (New England Biolabs). Then, the quality of the RNA samples was determined by measuring the optical density (OD) at 260 and 280 nm using a Thermo Scientific NanoDrop Lite spectrophotometer. qRT-PCR was performed using a KAPA SYBR Fast one-step qRT-PCR kit (Kapa Biosystems, Cape Town, South Africa). Each reaction mixture (final volume, 20 μL) comprised 10 μL of 2× KAPA SYBR Fast qPCR master mix (Universal), a final concentration of 200 nM for each forward and reverse primer, and 20 ng of RNA template in DNase/RNase-free water. Amplification was performed using a CFX96 Touch real-time PCR detection system (Bio-Rad Laboratories). The thermal cycling conditions were initial denaturation at 95°C for 3 min followed by 40 cycles of 95°C for 30 s, 53°C for 30 s, and 72°C for 30 s. A dissociation curve was created from a thermal profile of 95°C for 1 min, 55°C for 30 s, and 95°C (0.5°C/s). Each sample was assayed in triplicate. Gene expression levels relative to normal cells were analyzed using the threshold cycle (ΔΔ*CT*) method. The expression of caspase-3, caspase-6, and p53 was normalized against GAPDH, which was used as the internal control. The primers used in these experiments are shown in Table S3.

### Lactate dehydrogenase (LDH) detection.

The amount of LDH released from damaged cells was determined using a CytoTox96 kit (Promega, Madison, WI) and a spectrophotometer to measure the OD at 490 nm (OD_490_), according to the manufacturer’s instructions. The percentage of cytotoxicity was assessed by comparing the amount of LDH released from infected cells with the amount of LDH released from uninfected cells, which was considered a spontaneous release. The maximum release of LDH from uninfected cells was determined by lysing uninfected cells with 0.1% (vol/vol) Triton X-100. The following equation was used to calculate the percentage of toxicity: (OD_490_ experimental release − OD_490_ spontaneous release)/(OD_490_ maximum release − OD_490_ spontaneous release) × 100.

### Flow cytometry analysis.

Apoptotic cells were detected by double-staining with annexin V/propidium iodide (PI) using an FITC-annexin V apoptosis detection kit (BD Biosciences) according to the manufacturer’s instructions. Briefly, SH-SY5Y cells were infected with B. pseudomallei at an MOI of 20 for 10 h. Noninfected cells in the medium were included in the experiments as a negative control. After incubation, the cells were harvested by trypsinization and washed with ice-cold phosphate-buffered saline (PBS; pH 7.4). Then, 100 μL of the cell suspension (~1 × 10^5^ cells) was transferred to a 5-mL cell culture tube. The cells were stained by adding 5 μL each of fluorescein isothiocyanate (FITC)-annexin V and PI working solutions and incubated at 25°C for 15 min in the dark. Next, the preparation was diluted with 300 μL of 1× binding buffer, and then the cells were immediately fixed with 100 μL of 4% (wt/vol) paraformaldehyde. The stained SH-SY5Y cells were analyzed using an LSRFortessa flow cytometer (BD Biosciences).

### Bacterial growth analysis.

To determine the growth of B. pseudomallei strains, a single colony was inoculated into LB broth and cultured overnight at 37°C with shaking. The bacterial cultures were washed with PBS, and the density of the culture suspension was adjusted with LB broth to obtain approximately 1 × 10^8^ colony-forming unit per mL (CFU/mL). Then, 10 μL of the suspension was added to 10 mL of LB broth and incubated at 37°C with shaking. The culture was subjected to serial dilutions at 0, 2, 4, 6, 8, 10, and 24 h to determine the number of CFU.

### Invasion and intracellular replication assay.

*In vitro* invasion and intracellular replication assays were performed following infection of SH-SY5Y cells, as described above, with B. pseudomallei K96243 WT and Δ*bimA* mutant, as well as the Δ*bimA*::*bimA* strain. Briefly, to examine their ability to invade neuron cells, SH-SY5Y cells were infected with overnight cultures of B. pseudomallei strains at an MOI of 20. At 2 h post-infection, the extracellular bacteria were killed by adding fresh DMEM containing 250 μg/mL kanamycin (Sigma) and incubated at 37°C for 1 h. Then, the B. pseudomallei-infected cells were washed three times with PBS and lysed with 100 μL of 0.1% (vol/vol) Triton X-100. The invasion efficiency was determined by comparing the number of invading bacteria with the number of added bacteria and was determined according to the number of CFU. This was done by performing serial dilutions and then dropping 10 μL of each dilution onto LB agar plates and incubating the plates at 37°C for 24 to 48 h. Similarly, the number of intracellular bacteria was observed at 4, 6, 8, and 10 h post-infection.

### Determination of MNGC and plaque formation.

At 10 h post-infection, as described above, SH-SY5Y cells infected with the B. pseudomallei strains underwent Giemsa staining (Merck, Darmstadt, Germany), as described in our previous study (60). MNGC formation, which was defined as at least three nuclei in a cell, was evaluated in at least three fields of view at a magnification of 20× using a Nikon Ti2-E microscope. The percentage of MNGC formation was determined using the following formula: (number of nuclei in an MNGC/total number of nuclei counted) × 100. Each experiment was performed in duplicate, and a minimum of 1,000 nuclei per replicate were counted.

To observe plaque formation, the SH-SY5Y cells were infected with B. pseudomallei at an MOI of 1, and plaques were stained with 1% (wt/vol) crystal violet to enhance visualization at 20 h post-infection (25).

### Statistical analysis.

Each experiment was conducted in triplicate. Statistical analysis was performed using the GraphPad Prism 8 program (STATCON). Student’s *t* test of three independent experiments was used to determine the difference in quantitative data among different groups. All data in this study were presented as the mean ± standard error of the mean (SEM). A *P* value of ≤0.05 was considered statistically significant.

### Ethics statement.

All experiments and methods were performed per relevant guidelines and regulations. This project has been approved by the ethics committee of the Faculty of Tropical Medicine, Mahidol University, Bangkok, Thailand (reference no. MUTM-EXMPT 2022-004).

### Biosecurity aspects.

This project has been approved by the biosafety committee of the Faculty of Tropical Medicine, Mahidol University, Bangkok, Thailand (reference no. FTM-IBC 2021-039).
