# Supplementary figures and images for: In Vitro Roles of Burkholderia Intracellular Motility A (BimA) in Infection of Human Neuroblastoma Cell Line
Source: Microbiol Spectr. 2023 Jul 6;11(4):e01320-23. doi: 10.1128/spectrum.01320-23 (PMC10434047; doi:10.1128/spectrum.01320-23)

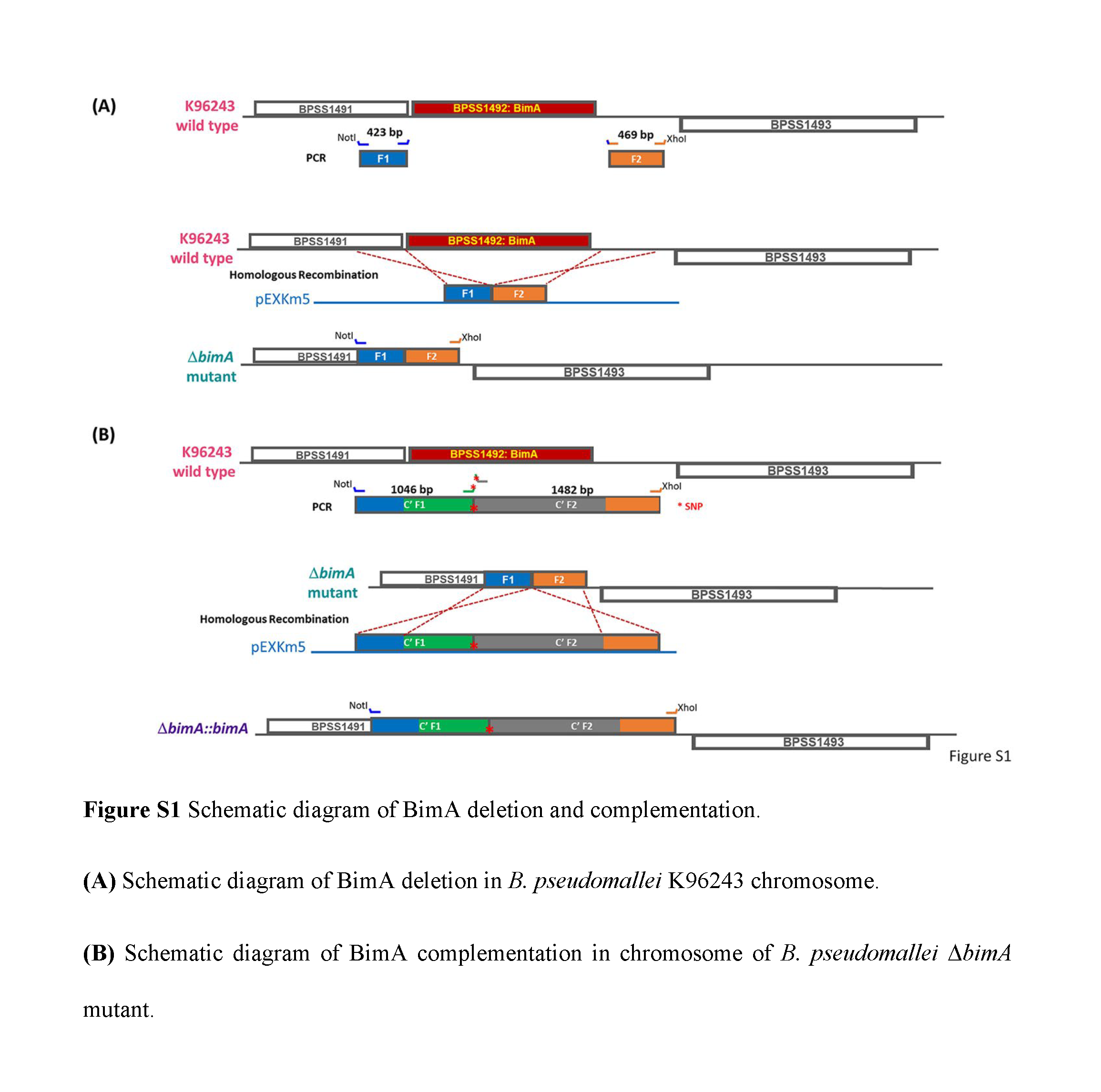

Supplement: Supplemental file 1 — Figure S1. Download spectrum.01320-23-s0001.tif, TIF file, 0.8 MB [file spectrum.01320-23-s0001.tif]

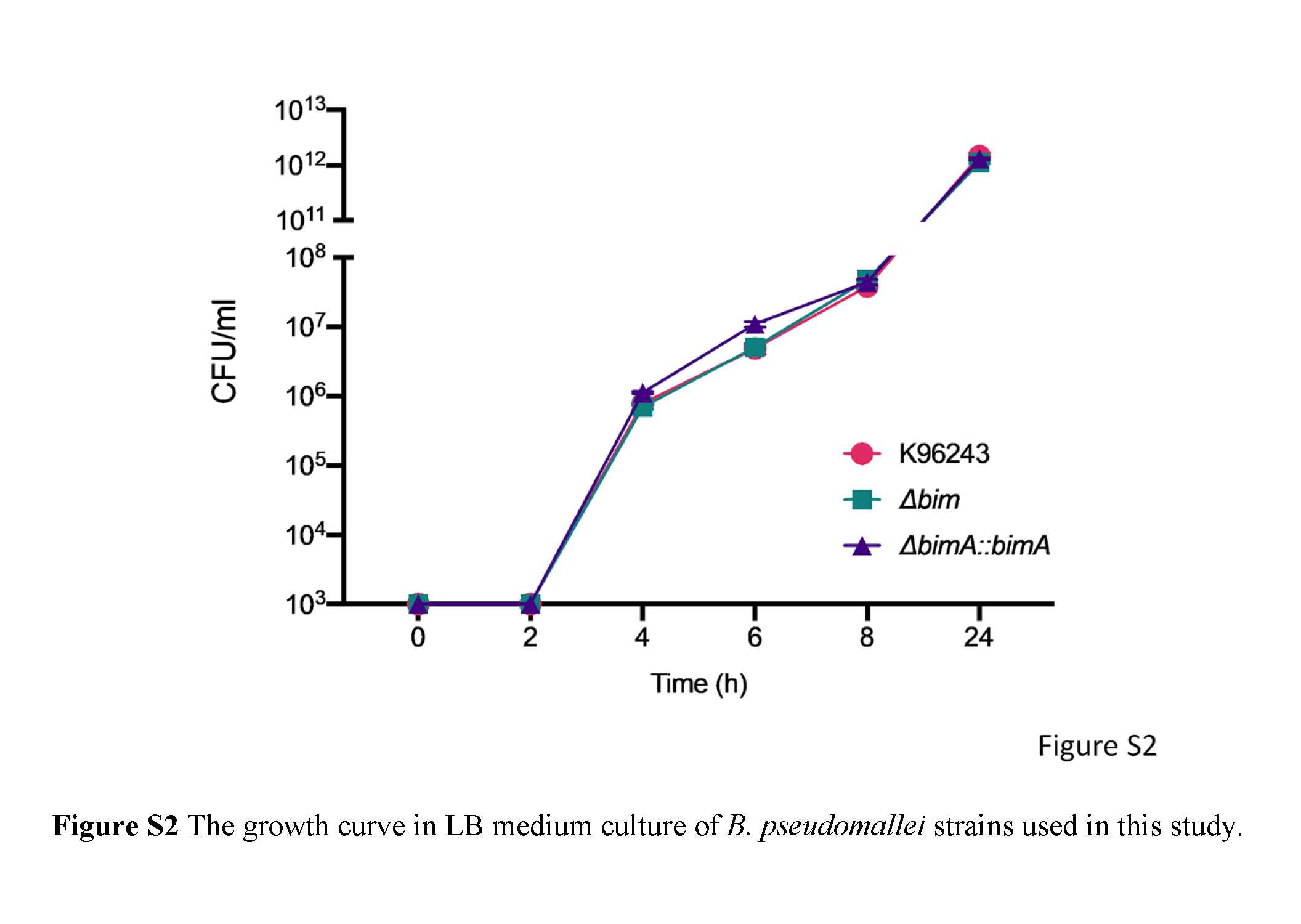

Supplement: Supplemental file 2 — Figure S2. Download spectrum.01320-23-s0002.tif, TIF file, 0.3 MB [file spectrum.01320-23-s0002.tif]

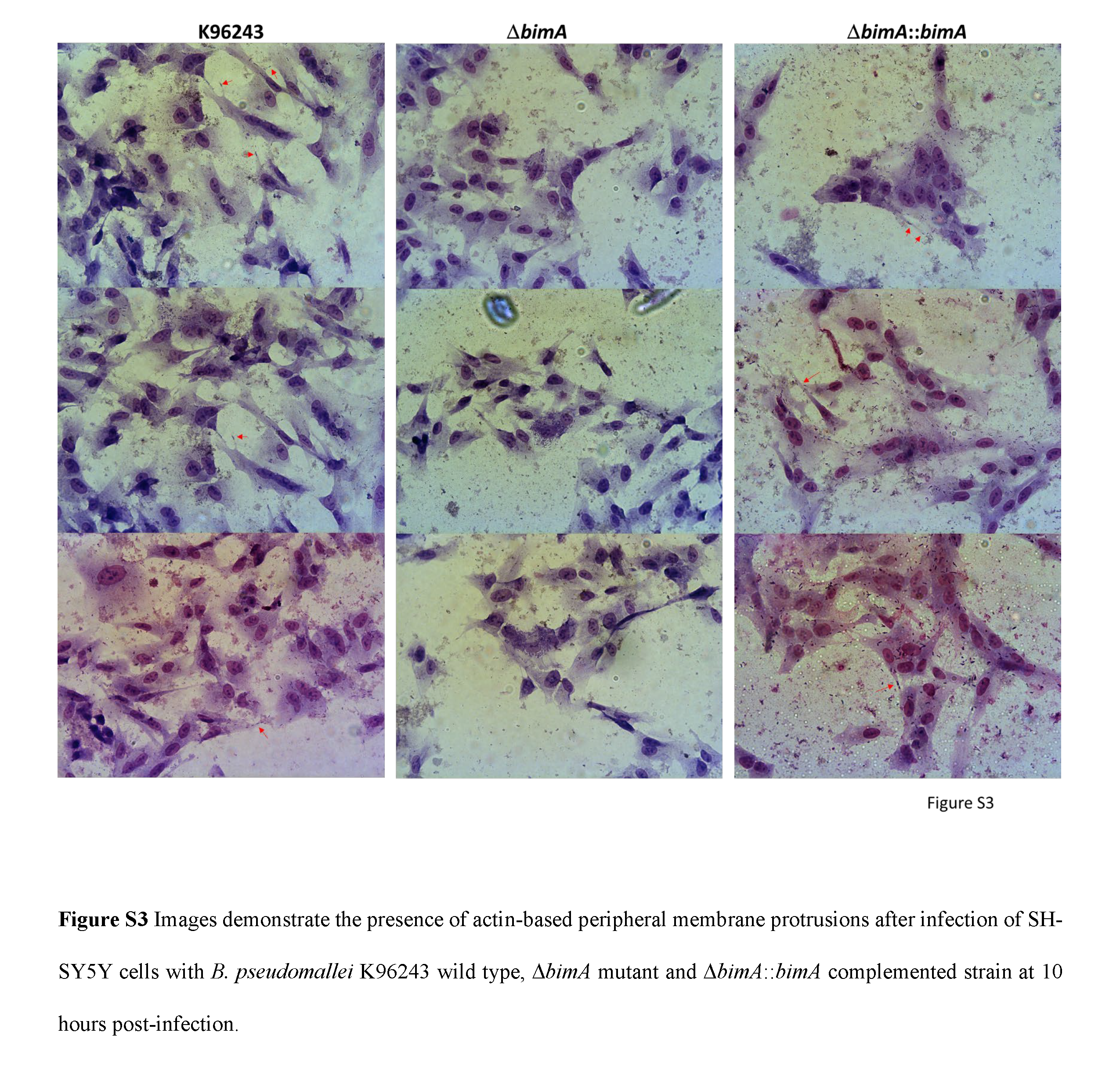

Supplement: Supplemental file 3 — Figure S3. Download spectrum.01320-23-s0003.tif, TIF file, 6.6 MB [file spectrum.01320-23-s0003.tif]

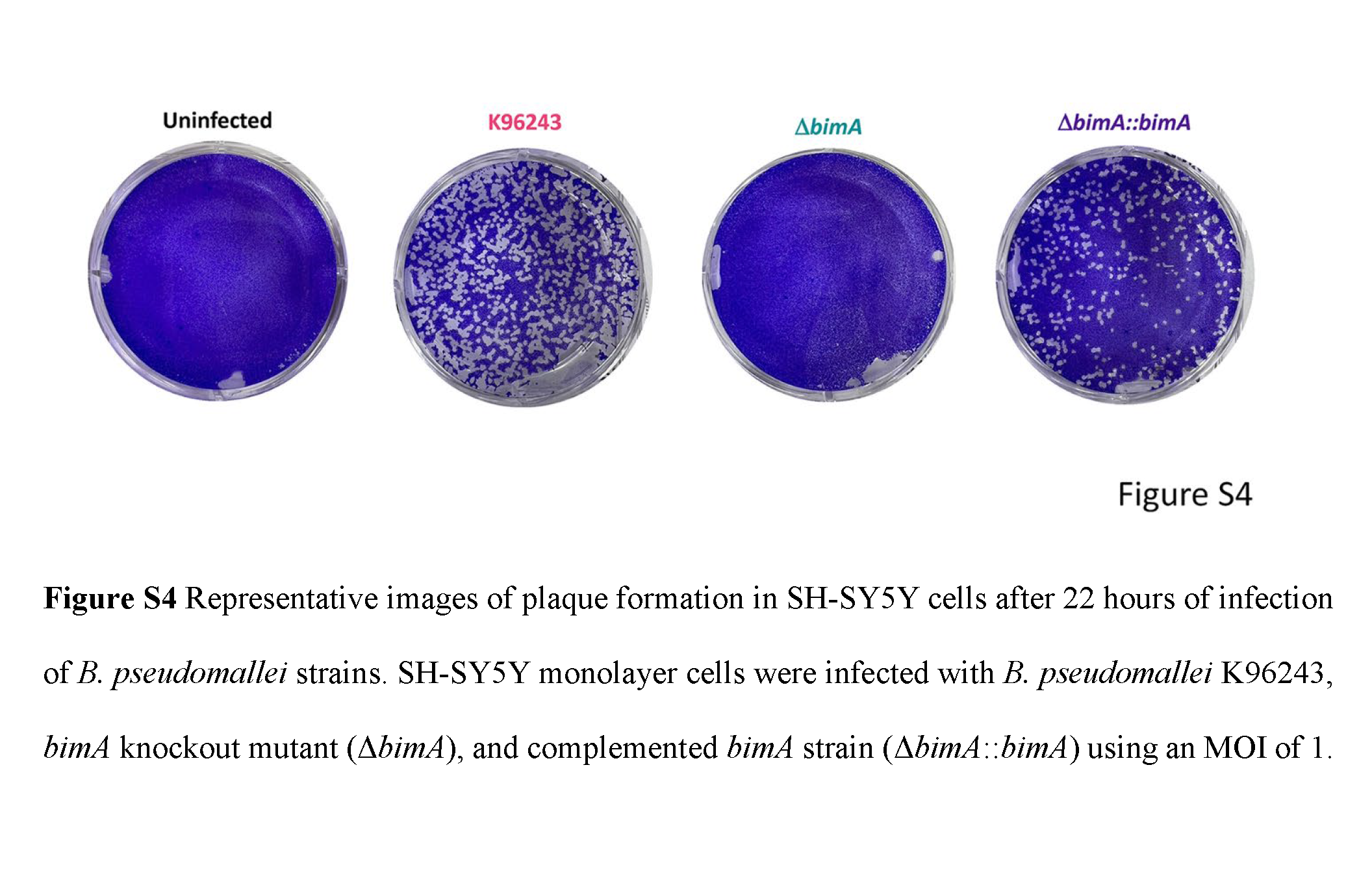

Supplement: Supplemental file 4 — Figure S4. Download spectrum.01320-23-s0004.tif, TIF file, 1.3 MB [file spectrum.01320-23-s0004.tif]
