# Supplementary material for: In Vitro Roles of Burkholderia Intracellular Motility A (BimA) in Infection of Human Neuroblastoma Cell Line
Source: Microbiol Spectr. 2023 Jul 6;11(4):e01320-23. doi: 10.1128/spectrum.01320-23 (PMC10434047; doi:10.1128/spectrum.01320-23)
Supplement: Supplemental file 7 — Table S3. Download spectrum.01320-23-s0007.docx, DOCX file, 0.04 MB [file spectrum.01320-23-s0007.docx]

Table S3

*In Vitro* Roles Played by *Burkholderia* Intracellular Motility A (BimA) During Infection of Human Neuroblastoma Cell Line

Niramol Jitprasutwit, Amporn Rungruengkitkun, Sanisa Lohitthai, Onrapak Reamtong, Nitaya Indrawattana, Nitat Sookrung, Thaniya Sricharunrat, Passanesh Sukphopetch, Narisara Chatratita, and Pornpan Pumirat*

***Correspondence:** Pornpan Pumirat: pornpan.pum@mahidol.edu

# Table S3: Primers used in this study.

| **Primer Name** | **Sequence (5′-3′)** |
| --- | --- |
| F1-BimA-NotI | tcgcgcgcggccgcCACGTTCTTCCACCCGACA |
| R1-BimA | TGGCAGTGACGTGGGATTAG |
| F2-BimA | CTAATCCCACGTCACTGCCAGCGACACGATGAAACTTCCG |
| R2-BimA-XhoI | tgcagactcgagGCTGCAGAACGCAGAATGAA |
| Seq-F-BimA | GCAAGTGCAGCGATTTCTGA |
| Seq-R-BimA | CGATCGTTCTCGGACGTGAC |
| oriT-F:  5'- | TCCGCTGCATAACCCTGCTTC |
| ori-T-R: 5' | CAGCCTCGCAGAGCAGGATTC |
| caspase 6 forward | TTCAGACGTTGACTGGCTTG |
| caspase 6 reverse | TTTCTGTTCACCAGCGTCAG |
| cas3 forward: | TGGTTCATCCAGTCGCTT TG |
| cas3 reverse | ATTCTGTTGCCACCTTTCG |
| p53 forward: | ACTAAGCGAGCACTGCCCAA |
| p53 reverse: | ATGGCGGGAGGTAGACTGAC |
| GAPDH forward | CTGGGCTACACTGAGCACC |
| GAPDH reverse: | AAGTGGTCGTTGAGG GCAATG |
