# Supplementary material for: In Vitro Roles of Burkholderia Intracellular Motility A (BimA) in Infection of Human Neuroblastoma Cell Line
Source: Microbiol Spectr. 2023 Jul 6;11(4):e01320-23. doi: 10.1128/spectrum.01320-23 (PMC10434047; doi:10.1128/spectrum.01320-23)
Supplement: Supplemental file 8 — Legends for Tables S1 to S3 and Figures S1 to S4. Download spectrum.01320-23-s0008.docx, DOCX file, 0.01 MB [file spectrum.01320-23-s0008.docx]

**SUPPLEMENTAL MATERIAL**

**Table S1** Identified proteins of neuroblastoma cells with/without *B. pseudomallei* K96243.

**Table S2** Identified proteins of neuroblastoma cells infected with *B. pseudomallei* K96243 and Δ*bimA* knockout mutant.

**Table S3** Primers used in this study.

**Figure S1**

**(A)** Schematic diagram of BimA deletion in *B. pseudomallei* K96243 chromosome.

**(B)** Schematic diagram of BimA complementation in chromosome of *B. pseudomallei* Δ*bimA* mutant.

**Figure S2** The growth curve in LB medium culture of *B. pseudomallei* strains used in this study.

**Figure S3** Images demonstrate the presence of actin-based peripheral membrane protrusions after infection of SH-SY5Y cells with *B. pseudomallei* K96243 wild type, Δ*bimA* mutant and Δ*bimA*::*bimA* complemented strain at 10 hours post-infection.

**Figure S4** Representative images of plaque formation in SH-SY5Y cells after 22 hours of infection of *B. pseudomallei* strains. SH-SY5Y monolayer cells were infected with *B. pseudomallei* K96243, *bimA* knockout mutant (Δ*bimA*), and complemented *bimA* strain (Δ*bimA*::*bimA*) using an MOI of 1.
